# Association of healthcare worker behaviors with coronavirus disease 2019 (COVID-19) risk during four pandemic periods and characteristics associated with high-risk behaviors

**DOI:** 10.1017/ash.2022.371

**Published:** 2023-01-17

**Authors:** Emily R. Egbert, Shaoming Xiao, Erica Prochaska, S. Omar Ali, Elizabeth Colantuoni, Avi Gadala, Danielle Koontz, Diana Zhong, Christina M. Schumacher, Anna C. Sick-Samuels, Amanda K. Debes, Aaron M. Milstone

**Affiliations:** 1 Division of Infectious Diseases, Department of Pediatrics, Johns Hopkins University School of Medicine, Baltimore, Maryland; 2 Department of International Health, Johns Hopkins Bloomberg School of Public Health, Baltimore, Maryland; 3 Department of Biostatistics, Johns Hopkins Bloomberg School of Public Health, Baltimore, Maryland; 4 Johns Hopkins Health System, Baltimore, Maryland; 5 Division of Infectious Diseases, Department of Medicine, Johns Hopkins University School of Medicine, Baltimore, Maryland; 6 Division of General Pediatrics, Department of Pediatrics, Johns Hopkins University School of Medicine, Baltimore, Maryland

## Abstract

In a large healthcare worker cohort, we quantified the association between behaviors and risk of coronavirus disease 2019 (COVID-19) during different pandemic phases, adjusting for prior infection and vaccination. Individual characteristics, including personal concerns, were associated with these behaviors. Public health messaging should target high-risk populations and behaviors as the pandemic evolves.

Transmission of severe acute respiratory coronavirus virus 2 (SARS-CoV-2) occurs primarily through respiratory particles from infected individuals.^1^ Proposed infection prevention measures include the use of face masks, outdoor gatherings, and increased ventilation; however, as the pandemic has evolved, few studies have evaluated how individual behaviors affect infection risk or which factors affect individual choices to engage in certain behaviors. We examined the association of healthcare worker (HCW) behaviors with coronavirus disease 2019 (COVID-19) risk during 4 pandemic periods to identify characteristics associated with high-risk behaviors.

## Methods

### Study design and participants

We performed a longitudinal cohort of HCWs within the Johns Hopkins Health System to determine the seroprevalence of spike antibodies to SARS-CoV-2. Every 2–4 months, participants provided blood samples and completed surveys.^2,3^ This analysis included 2 electronic behavior surveys, completed in the fall of 2021 (survey A) and the spring of 2022 (survey B), that detailed behaviors during 4 periods between February 2021 and January 2022 (Supplementary Fig. 1). This study was approved by the Johns Hopkins University Institutional Review Board.

### Data collection

The primary outcome was presence of infection defined as a positive SARS-CoV-2 polymerase chain reaction, SARS-CoV-2 antigen, or IgG antibody level (preceding vaccination), regardless of workplace or non-workplace exposure. Results were grouped into 4 periods to align with temporally reported behaviors. Behaviors, participant demographic and clinical characteristics, COVID-19 exposures, prior COVID-19, and personal concerns about COVID-19 were collected from survey responses. ZIP codes were grouped by county and aligned with county mask mandates as of December 2021.

### Analysis

Poisson regression models were used to estimate the relative risk of infection comparing participants who did versus did not engage in each behavior, adjusting for study period. Up to 4 behavior assessments per participant were included. Sensitivity analyses adjusted for prior infection and receipt of vaccine booster before December 2021. Poisson regression models were also used to estimate the relative risk of engaging in a specific behavior with participant demographic and clinical characteristics and risk perceptions during period 4. Poisson regression models included robust variance estimates. Statistical significance was based on estimated 95% confidence intervals (CIs) for the relative risk (RR).

## Results

Of 1,987 participants who gave consent, 1,597 (80%) completed survey A or survey B; 1,375 (86%) completed survey A in fall 2021 and 1,125 (70%) completed survey B in March 2022. Overall, 5,250 behavior assessments represented 4 periods. Of the 1,597, the median age was 43.2 (interquartile range [IQR], 35.4–54.6), 81.7% were female, and 82.3% were white (96.0% were non-Hispanic/Latino) (Supplementary Table 1). Moreover, 33.3% were clinicians and 33.5% were nursing staff. As of February 2022, 23.9% had been infected with SARS-CoV-2.

Across all study periods, 89.7% of responses reported wearing masks indoors, 37.2% dined and drank indoors, and 21.1% attended large gatherings or events (Table 1). After adjusting for study period, wearing masks indoors was associated with reduced infection risk, whereas dining and drinking indoors at restaurants or bars and attending large gatherings or events were associated with increased risk of SARS-CoV-2 infection. Other reported behaviors were not associated with risk of infection, including wearing a mask outdoors, traveling out of state by car, or plane, or taking public transportation. Similar association of behaviors and SARS-CoV-2 infection risk were found in sensitivity analyses accounting for (1) prior infection and study period (Supplementary Table 2) and (2) the impact of COVID-19 vaccine booster prior to December 2021 and prior infection (Supplementary Table 3).

**Table 1.** Association of Reported Behaviors With Risk of COVID-19 Infection in 1,597 Participants Completing 5,250 Behavior Assessments

Risk Behavior	No. (%) Reporting Behavior^a^	No. (%) with COVID-19 Infection^b^ Who Reported Performing Behavior	No. (%) with COVID-19 Infection^b^ Who Reported Not Performing Behavior	Adjusted Relative Risk (IQR)^c^ of COVID-19 Infection
Wear mask indoors	4,708 (89.7)	158 (3.4)	24 (4.4)	0.55 (0.37–0.82)
Wear mask outdoors	2,017 (38.4)	74 (3.7)	108 (3.3)	1.00 (0.76–1.32)
Wear mask indoors with family and friends	1,829 (34.8)	48 (2.6)	134 (3.9)	0.75 (0.55–1.02)
Dine/drink indoors at restaurants/bars	1,952 (37.2)	79 (4.0)	103 (3.1)	1.32 (1.01–1.74)
Attend large gatherings/events	1,106 (21.1)	49 (4.4)	133 (3.2)	1.44 (1.06–1.96)
Travel out-of-state by car	3,380 (64.4)	111 (3.3)	71 (3.8)	1.01 (0.76–1.34)
Travel out-of-state by plane	1,883 (35.9)	76 (4.0)	106 (3.1)	1.25 (0.95–1.65)
Travel internationally	913 (14.3)	22 (2.6)	160 (3.0)	1.12 (0.73–1.72)
Take public transportation	1,073 (16.8)	30 (2.9)	152 (2.9)	1.09 (0.76–1.57)

Note. IQR, interquartile range.

a Denominator based on number of behavior assessments completed during all periods (n = 5,250).

b Participants could contribute multiple infections in different periods.

c Adjusted for period and reported with 95% Confidence Interval that was calculated using robust error variance.

The association of participant characteristics with behaviors that contribute to COVID-19 risk were assessed in 1,125 participants who completed survey B during period 4, the SARS-CoV-2 omicron variant wave (Table 2). Compared to participants aged ≤29 years, participants aged 30–39 years were more likely to wear masks indoors. Participants aged ≥30 years were less likely to engage in dining and drinking indoors at restaurants or bars and attending large gatherings or events compared to those aged ≤29 years. Living in a county with a mask mandate increased participants’ reported likelihood of wearing a mask indoors and decreased their likelihood of dining and drinking indoors at restaurants or bars. Of the underlying conditions reported, only immunocompromised status increased the likelihood of masking indoors. Prior COVID-19 was associated with reduced likelihood of wearing a mask indoors and increased likelihood of dining and drinking indoors at restaurants or bars and attending large gatherings or events.

**Table 2.** Association of Participant^a^ Characteristics With Behaviors Reported During Omicron Wave (December 2021 through January 2022)

Clinical Characteristics	Wear a Mask Indoors	Dine/Drink Indoors at Restaurants/Bars	Attend Large Gatherings/Events
**Age group**			
≤29 y	Reference	Reference	Reference
30–39 y	1.10 (1.01–1.20)^2^	0.52 (0.40–0.68)^2^	0.53 (0.36–0.79)^b^
40–49 y	1.04 (0.96–1.14)	0.75 (0.59–0.97)	0.58 (0.40–0.86)
50–59 y	1.06 (0.97–1.15)	0.72 (0.56–0.93)	0.57 (0.38–0.85)
≥60 y	1.08 (0.99–1.18)	0.60 (0.44–0.81)	0.47 (0.29–0.75)
**Sex**			
Female	Reference	Reference	Reference
Male	1.03 (0.99–1.06)	1.03 (0.85–1.26)	0.88 (0.64–1.22)
**Race**^**c**^			
White	Reference	Reference	Reference
Asian	1.06 (1.03–1.09)	1.12 (0.88–1.44)	0.98 (0.65–1.48)
African American	1.06 (1.02–1.10)	1.46 (1.11–1.92)	2.05 (1.43–2.96)
Other^d^	0.99 (0.87–1.12)	0.85 (0.45–1.60)	0.68 (0.24–1.99)
**Ethnicity**			
Not Hispanic/Latino	Reference	Reference	Reference
Hispanic/Latino	1.02 (0.96–1.09)	0.86 (0.55–1.33)	0.67 (0.32–1.44)
**Other characteristics**			
Mask mandate in county of primary residence as of 12/31/2021^e^	1.13 (1.05–1.21)	0.78 (0.63–0.96)	0.80 (0.57–1.12)
Diabetes^f^	1.04 (0.98–1.10)	1.17 (0.78–1.76)	1.24 (0.67–2.31)
Obesity^f^	1.02 (0.98–1.06)	0.85 (0.66–1.10)	0.80 (0.54–1.19)
Smoking (current/former)^f^	0.97 (0.87–1.08)	0.85 (0.51–1.44)	0.79 (0.35–1.79)
Immune deficiency, cancer, transplant^f^	1.07 (1.05–1.09)	0.97 (0.62–1.51)	0.68 (0.30–1.56)
COVID-19 infection prior to period 4^g^	0.91 (0.85–0.97)	1.39 (1.14–1.70)	1.91 (1.45–2.52)
Booster before 12/1/2021	1.17 (1.11–1.23)	0.67 (0.57–0.79)	0.60 (0.47–0.76)

a 1,125 participants who completed survey B were included in this analysis.

b Risk ratios with 95% Confidence Interval were calculated using robust error variance.

c Participants were excluded from this variable who reported nonbinary sex (n = 3).

d Participants who reported American Indian/Alaskan Native, Native Hawaiian/Pacific Islander and other race were grouped as other race due to small sample size.

e Based on participants’ self-reported ZIP code of primary residence and the mask mandate for the county encompassing that ZIP code as of December 31, 2021.

f Underlying conditions based on self-report of participants during period 4. Conditions contributing to immunocompromised state were grouped together as immune deficiency, cancer, or transplant.

g Includes participants who tested PCR positive for SARS-CoV-2 or who self-reported having tested positive for SARS-CoV-2 before period 4.

The association of participant concerns and behaviors reported in survey A were correlated with behaviors during period 4. Those who were concerned about exposing others, disruption, or personal illness were more likely to wear a mask indoors, were less likely to dine and drink indoors at restaurants or bars and were less likely to attend large gatherings or events (Supplementary Table 4).

## Discussion

This analysis quantified the association between behaviors and risk of COVID-19 among a cohort of HCW during different phases of the pandemic. Consistent with public health messaging and other studies, wearing a mask indoors was associated with reduced COVID-19 risk.^4–6^ Few studies have previously explored the relationship between behaviors that increase exposure to COVID-19 and individual infection risk.^7^ Dining and drinking indoors at restaurants or bars or attending large gatherings or events were associated with increased infection risk. These results remained robust even when adjusting for prior infection.

Given limited data on personal characteristics or beliefs that may influence why people choose to engage in behaviors that affect risk of exposure to COVID-19, a strength of this study was the assessment of factors associated with reporting engagement in different behaviors. Age, select underlying conditions, receipt of booster dose of vaccine, and prior COVID-19 infection, correlated with behaviors during the SARS-CoV-2 ο (omicron) surge. Additionally, place of residence was associated with participants’ behaviors, such that participants who resided in a ZIP code without a mask mandate reported being less likely to wear a mask indoors and more likely to dine indoors and to attend large gatherings. This finding supports the need for consistent messaging and policies from local health departments to limit infection risk.

These data are also consistent with the current literature showing that perception of risk may influence precaution behavior.^8^ Participants who experienced prior infection were less likely to wear masks in indoor spaces, more likely to dine and drink indoors, and more likely to attend large gatherings. The durability of protection from COVID-19 following infection is uncertain, as is the infection risk from new variants and decaying immunity despite prior infection. Personal concerns had the most ubiquitous effect on participant behavior, increasing the likelihood of masking indoors as well as decreasing the risk of dining and drinking indoors and attending large gatherings.

This study of HCWs with likely high health literacy has limited diversity. Although the findings regarding behaviors may not be widely generalizable, the findings are potentially generalizable to a broader population of healthcare workers.^9^ Few infections occurred during the first 3 study periods, but the rate of infections is consistent with the national COVID-19 incidence. We were unable to control for COVID-19 exposures. Recall and social desirability bias may have affected responses. Despite these limitations, these findings demonstrate the consistent association between behaviors and infection risk and that certain characteristics and beliefs are associated with engaging in these behaviors. As the pandemic evolves, people will continue to make personal decisions about personal risk of infection and partaking or avoiding behaviors associated with exposure and infection. Future studies should explore how to optimize consistent and factual public health messaging so help inform the chosen behaviors of individuals.

## Supplementary material

For supplementary material accompanying this paper visit https://doi.org/10.1017/ash.2022.371. click here to view supplementary material
