# Supplementary material for: Association of healthcare worker behaviors with coronavirus disease 2019 (COVID-19) risk during four pandemic periods and characteristics associated with high-risk behaviors
Source: Antimicrob Steward Healthc Epidemiol. 2023 Jan 17;3(1):e16. doi: 10.1017/ash.2022.371 (PMC9879855; doi:10.1017/ash.2022.371)
Supplement: Supplementary file 1 [file S2732494X22003710sup001.docx]

Supplemental Materials for “Association of Healthcare Worker Behaviors with COVID-19 Infection Risk during Four Pandemic Periods and Characteristics Associated with High Risk Behaviors”

Emily R. Egbert, MAT, MPH^1^ **^*^**, Shaoming Xiao, MSPH^1^ **^*^**, Erica Prochaska, MD^1^, S. Omar Ali, PhD MPH^2#^, Elizabeth Colantuoni, PhD, ScM^3,^ Avi Gadala, PhD, MS^4^, Danielle Koontz, MAA, MS^1^, Diana Zhong, MD^5^, Christina M. Schumacher, PhD^6^, Anna C. Sick-Samuels MD MPH^1^, Amanda K. Debes, PhD, MS^2^, Aaron M. Milstone, MD, MHS^1^

*****authors contributed equally (E.E. and S.X.)

Includes:

Supplemental Figure 1: Timeline of study periods included in analysis and surveys conducted.

Supplemental Table 1: Demographics and clinical characteristics of participants who completed survey(s)

Supplemental Table 2: Association of reported behaviors with risk of COVID-19 infection adjusted for study period and prior infection in participants completing a survey (N = 1597)

Supplemental Table 3: Association of reported behaviors and the risk of COVID-19 infection during the Omicron surge (Period 4, December 2021 to February 2022) adjusting for receipt of COVID-19 vaccine booster and prior infection in participants completing a survey (N = 1125)

Supplemental Table 4: Association of participant^1^ concerns^2^ and behaviors reported in the Fall of 2021 (September through November 2021) with corresponding behaviors during the Winter of 2021 (December 2021 through January 2022)


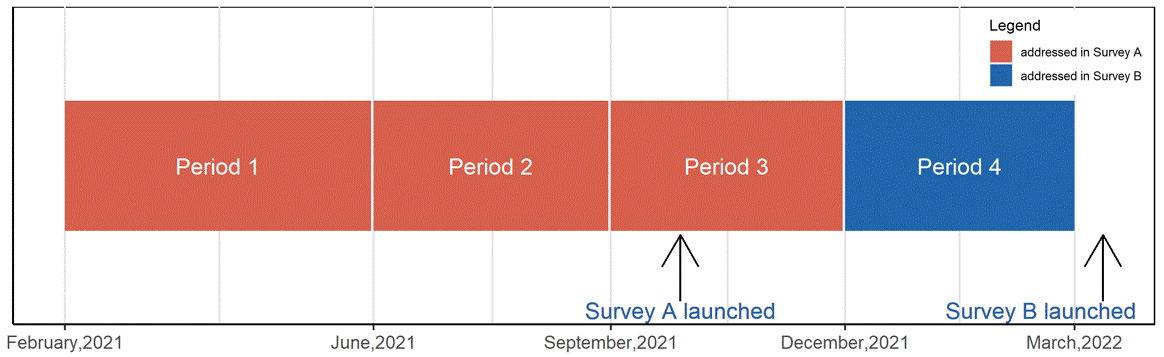


**Supplemental Figure 1**: Timeline of study periods included in analysis and surveys conducted. Survey A was launched on Sep 28, 2021 and included questions about behaviors during February/March 2021 (second COVID wave), June/July 2021 (low COVID incidence), and September to November 2021 (Delta wave). Survey B was launched on March 11, 2022 and included questions about behaviors during December 2021/January 2022 (Omicron wave). Infections were identified and grouped into the following outcome periods: February-May 2021 (Period 1), June-August 2021 (Period 2), September-November 2021 (Period 3), December 2021-February 2022 (Period 4).

**Supplemental Table 1:** Demographics and clinical characteristics of participants who completed survey(s)

|  | Survey A^1^  (N = 1375) | Survey B^1^  (N = 1125) | Both Surveys  (N = 903) | Any survey  (N = 1597) |
| --- | --- | --- | --- | --- |
| Age group: n (%) |  |  |  |  |
| <= 29 | 132 (9.6) | 82 (7.3) | 71 (7.9) | 143 (9.0) |
| 30-39 | 443 (32.2) | 320 (28.4) | 261 (28.9) | 502 (31.4) |
| 40-49 | 341 (24.8) | 292 (26.0) | 236 (26.1) | 397 (24.9) |
| 50-59 | 283 (20.6) | 268 (23.8) | 210 (23.3) | 341 (21.3) |
| >=60 | 176 (12.8) | 163 (14.5) | 125 (13.8) | 214 (13.4) |
| Sex^2^ |  |  |  |  |
| Female | 1128 (82.0) | 913 (81.2) | 737 (81.6) | 1304 (81.7) |
| Male | 244 (17.8) | 209 (18.6) | 165 (18.3) | 288 (18.0) |
| Race |  |  |  |  |
| American Indian/Alaskan Native | 1 (0.1) | 1 (0.1) | 0 | 2 (0.1) |
| Asian | 140 (10.2) | 117 (10.4) | 87 (9.7) | 170 (10.6) |
| Black/African American | 62 (4.5) | 56 (4.9) | 40 (4.4) | 78 (4.9) |
| Native Hawaiian/Pacific Islander | 4 (0.3) | 3 (0.3) | 3 (0.3) | 4 (0.3) |
| White | 1142 (83.0) | 928 (82.5) | 755 (83.6) | 1315 (82.3) |
| Other | 26 (1.9) | 20 (1.8) | 18 (2.0) | 28 (1.8) |
| Ethnicity |  |  |  |  |
| Hispanic/Latino | 56 (4.1) | 46 (4.1) | 38 (4.2) | 64 (4.0) |
| Not Hispanic/Latino | 1319 (95.9) | 1079 (95.9) | 871 (95.8) | 1533 (96.0) |
| Job Role |  |  |  |  |
| Administrative/Research | 214 (15.6) | 176 (15.7) | 148 (16.4) | 242 (15.2) |
| Allied Health Worker | 182 (13.2) | 142 (12.6) | 124 (13.7) | 200 (12.5) |
| Clinicians | 442 (32.1) | 371 (33.0) | 281 (31.1) | 532 (33.3) |
| Nursing | 463 (33.7) | 367 (32.6) | 295 (32.7) | 535 (33.5) |
| Support Staff | 74 (5.4) | 69 (6.1) | 55 (6.1) | 88 (5.5) |
| Ever infected with COVID-19 prior to end of the study period ^3^ | 332 (24.2) | 261 (23.1) | 212 (23.5) | 381 (23.9) |
| Infected with COVID-19 prior to study period^3^ | 137 (10.0) | 102 (9.1) | 92 (10.2) | 147 (9.2) |
| Infected with COVID-19 during study period ^3^ | 195 (14.2) | 159 (14.1) | 120 (13.3) | 234 (14.7) |

^1^ Survey A included risk assessments for each of the following time periods: February – March 2021, June – July 2021, September –November 2021. Survey B included a risk assessment for December 2021 – January 2022

^2^ Among participants in any survey, 5 did not report sex, 3 of them completed survey A, 3 of them completed survey B and 1 completed both surveys.

^3^ Defined as PCR-confirmed infection, self-reported infection, or IgG positive before receiving a COVID-19 vaccine

**Supplemental Table 2:** Association of reported behaviors with risk of COVID-19 infection adjusted for study period and prior infection in participants completing a survey (N = 1597)

| Behavior | Relative Risk^1^ of COVID-19 infection |
| --- | --- |
| Wear mask indoors | 0.48 (0.33 - 0.72) |
| Wear mask outdoors | 0.95 (0.72 - 1.25) |
| Wear mask indoors with family and friends | 0.72 (0.53 - 0.99) |
| Dine/drink indoors at restaurants/bars | 1.41 (1.07 - 1.86) |
| Attend large gatherings/events | 1.58 (1.16 - 2.16) |
| Travel out-of-state by car | 1.06 (0.79 - 1.41) |
| Travel out-of-state by plane | 1.33 (1.00 - 1.76) |
| Travel internationally | 1.13 (0.74 - 1.74) |
| Take public transportation | 1.13 (0.78 - 1.63) |

^1^ Adjusted for time period and prior infection, and reported with 95% Confidence Interval that was calculated using robust error variance

**Supplemental Table 3:** Association of reported behaviors and the risk of COVID-19 infection during the Omicron surge (Period 4, December 2021 to February 2022) adjusting for receipt of COVID-19 vaccine booster and prior infection in participants completing a survey (N = 1125)

| Behavior | Unadjusted Relative Risk^1^ of COVID-19 infection^2^ during Period 4 | Relative Risk^1^ of COVID-19 infection^2^ adjusted for booster dose during Period 4 | Relative Risk^1^ of COVID-19 infection^2^ adjusted for booster dose and prior infection during Period 4 |
| --- | --- | --- | --- |
| Wearing masks indoors | 0.48 (0.31 - 0.76) | 0.55 (0.34 - 0.88) | 0.50 (0.32 - 0.79) |
| Wearing masks outdoors | 1.09 (0.80 - 1.50) | 1.14 (0.83 - 1.56) | 1.07 (0.78 - 1.46) |
| Wearing masks indoors with family and friends | 0.80 (0.56 - 1.14) | 0.83 (0.58 - 1.18) | 0.80 (0.57 - 1.14) |
| Dine/drink indoors at restaurants/bars | 1.34 (0.98 - 1.84) | 1.28 (0.93 - 1.76) | 1.35 (0.98 - 1.84) |
| Attend large gatherings/events | 1.40 (0.98 - 2.00) | 1.33 (0.91 - 1.92) | 1.47 (1.02 - 2.12) |
| Travel out-of-state by car | 1.06 (0.77 - 1.46) | 1.07 (0.78 - 1.47) | 1.14 (0.83 - 1.56) |
| Travel out-of-state by plane | 1.15 (0.84 - 1.59) | 1.14 (0.83 - 1.57) | 1.24 (0.90 - 1.70) |
| Travel internationally | 1.06 (0.64 - 1.75) | 1.05 (0.63 - 1.72) | 1.05 (0.64 - 1.72) |
| Take public transportation | 1.05 (0.69 - 1.61) | 1.04 (0.67 - 1.59) | 1.07 (0.70 - 1.63) |

^1^ Risk ratios with 95% Confidence Interval was calculated using robust error variance

^2^ Infections in period 4 = 137 (12.2%)

**Supplemental Table 4:** Association of participant^1^ concerns^2^ and behaviors reported in the Fall of 2021 (September through November 2021) with corresponding behaviors during the Winter of 2021 (December 2021 through January 2022)

|  | Wear a mask indoors | Dine/drink indoors at restaurants/bars | Attend large gatherings/events |  |
| --- | --- | --- | --- | --- |
| Any reported concerns |  |  |  |  |
| No | Ref | Ref | Ref |  |
| Yes | 1.22 (1.12 - 1.33)^3^ | 0.50 (0.42 - 0.60) ^3^ | 0.44 (0.33 - 0.57) ^3^ |  |
| I don’t know | 1.09 (0.93 - 1.28) | 0.62 (0.40 - 0.97) | 0.55 (0.27 - 1.10) |  |
| Concern about exposing others | 1.18 (1.09 - 1.27) | 0.58 (0.47 - 0.70) | 0.49 (0.36 - 0.67) |  |
| Concern about disruption | 1.09 (1.05 - 1.13) | 0.65 (0.54 - 0.79) | 0.49 (0.36 - 0.68) |  |
| Concern about personal significant illness | 1.11 (1.05 - 1.16) | 0.73 (0.60 - 0.89) | 0.58 (0.43 - 0.79) |  |
| ^1^ 903 participants who completed Survey A and B and were included in this analysis  ^2^ Participants were asked whether they were concerned about getting infected with COVID-19, and concerns were grouped into three categories: concern about exposing others (exposing coworkers and/or patients, a susceptible family member who may be at risk of severe disease, or a susceptible family member who may not be able to go to school/work), concern about disruption (not being able to work or care for dependents), and concern about personal significant illness (getting hospitalized, severe illness, or persistent symptoms).  ^3^ Risk ratios with 95% Confidence Interval was calculated using robust error variance. | | | | |
